# Locally advanced hypopharyngeal squamous cell carcinoma: single-institution outcomes in a cohort of patients curatively treated either with or without larynx preservation*

**DOI:** 10.1590/0100-3984.2015.0018

**Published:** 2016

**Authors:** Isabel Reis, Artur Aguiar, Cristiana Alzamora, Carolina Ferreira, Vera Castro, André Soares, Marisa Lobão

**Affiliations:** 1 MDs, Radiation Oncologists, Radiation Oncology Department, Instituto Português de Oncologia do Porto Francisco Gentil (IPO-Porto), Porto, Portugal.; 2 MDs, Radiation Oncology Residents, Radiation Oncology Department, Instituto Português de Oncologia do Porto Francisco Gentil (IPO-Porto), Porto, Portugal.

**Keywords:** Hypopharynx, Outcomes, Surgery, Radiotherapy

## Abstract

**Objective:**

The present study was aimed at describing a single-institution experience in the curative treatment of patients diagnosed with locally advanced hypopharyngeal squamous cell carcinoma.

**Materials and Methods:**

Data concerning all patients treated for locally advanced hypopharyngeal squamous cell carcinoma between January 2006 and June 2012 were reviewed.

**Results:**

A total of 144 patients were included in the present study. The median follow-up period was 36.6 months. Median survival was 26 months, and 2-year and 5-year overall survival rates were, 51% and 30.5%, respectively. Median recurrence-free survival was 18 months and 2-year and 5-year recurrence-free survival rates were 42.8% and 28.5%, respectively.

**Conclusion:**

The outcomes in the present series are in line with the literature.

## INTRODUCTION

It has become evident that the behavior of cancers arising in the hypopharynx is significantly different from those arising in other head and neck subsites, despite their similar histological appearance. The worse prognosis of hypopharynx squamous cell carcinoma (SCC) is related to several factors, namely: the high propensity to lymphatic and systemic spread; predisposition to development of second malignancies; frequent advanced stage at presentation; frequent association with major alcohol abuse history and associated medical comorbidities; and frequent nutritional depletion.

In the locally advanced setting, the therapeutic decision still raises controversy and, frequently, appropriate treatment strategy requires a multidisciplinary approach.

For anatomical reasons, a primary surgical approach typically requires a total laryngectomy. Therapeutic strategies aimed at larynx preservation include: partial pharyngolaryngectomy; radiotherapy (RT) alone; concomitant radiochemotherapy (RT/CT); induction chemotherapy (CT) followed by RT or RT/CT in good responders; and the combination of RT with anti-epidermal growth factor receptor (anti-EGFR) therapy.

So far, no randomized trial has compared definitive RT/ CT with primary surgical approach specifically in cases of hypopharyngeal cancer, but a randomized trial developed by the European Organization for Research and Treatment of Cancer (EORTC) has compared induction CT followed by RT with total laryngectomy plus adjuvant RT, and the results showed that the larynx preserving strategy did not cause significant worsening in disease management nor jeopardized the patients' overall survival (OS)^(1)^.

The present study was aimed at reporting follow-up data concerning patients with locally advanced hypopharynx SCC treated with curative intent either with or without larynx preservation approach in a single institution and comparing it with results of other published studies.

## MATERIALS AND METHODS

### Patients

Clinical records of all patients who presented with hypopharynx SCC between January 2006 and June 2012 in the authors' department were retrospectively reviewed.

Overall, 177 patient records were reviewed, and 144 patients met the inclusion criteria and were therefore included in the present study. The exclusion criteria were the following: disease stages I and II (*n* = 6); patients undergoing palliative RT (*n* = 3); presence of second primary tumors (*n* = 5); presence of recurrent tumors (*n* = 1); synchronous tumors (*n* = 2); presence of distant metastases (*n* = 1); and patients that did not complete RT due to complications (*n* = 10) or because they refused/abandoned the treatment (*n* = 5).

### Treatment

Before starting the treatment, all the patients were evaluated by a multidisciplinary head and neck tumor board.

Sixty-three patients (43.8%) were submitted to surgery followed by adjuvant RT. Intensity modulated radiation therapy (IMRT) and volumetric modulated arc therapy (VMAT) treatments were performed in 12 of those patients (19.0%), while the others received three-dimensional (3D) conformal RT. The median prescribed dose to surgical bed and lymph node areas with extracapsular extension was 66 Gy. In the presence of residual tumor, a dose of 70 Gy was prescribed to that area. The region with higher risk of recurrence corresponding to the area adjacent to the surgical bed suspected of possible subclinical spread plus lymph node levels adjacent to the ones involved, received 60 Gy. The low-risk region, corresponding to the lymph node levels at lower risk of subclinical infiltration, received 50 Gy. Median overall treatment time was 46 (41-57) days. Thirty-nine (61.9%) patients received CT concomitantly with adjuvant RT, consisting of cisplatin in 37 patients (94.9%) and carboplatin in 2 patients (5.1%). Only one patient in this group received induction CT (1.6%), consisting of cisplatin + 5-fluorouracil.

The other 81 patients (56.3%) received radical RT integrated in an organ preservation approach. IMRT/VMAT treatments were performed in 19 of such patients (23.5%), while the others received 3D conformal RT. Grossly visible primary tumor and metastatic lymphadenopathy demonstrated at imaging or physical examination received 70 Gy. The region with the higher risk of recurrence, corresponding to the area adjacent to the primary tumor suspected of possible subclinical spread plus the lymph node levels adjacent to the ones involved, received 60 Gy. The low-risk region, corresponding to the lymph node levels at lower risk of subclinical infiltration, received 50 Gy. Median overall treatment time was 49 (44-74) days. Most patients (*n* = 73; 90.1%) received concurrent CT which consisted of cisplatin in 60 patients (74.1%), cisplatin + 5-fluorouracil in 1 patient (1.2%), and carboplatin in 4 patients (4.9%). Three patients in this group have not received CT as they refused or did not have clinical conditions to endure the treatment. Induction CT was given to 25 patients (30.9%) in the radical RT group and consisted of cisplatin + 5-fluorouracil in 13 patients (52.0%), and doxetaxel + cisplatin + 5-fluorouracil in 12 patients (48.0%).

As regards RT planning, the patients were immobilized in supine position with a thermoplastic head-and-neck mask. Computed tomography scan was performed for each patient in the treatment position with slice thickness of 2.5 mm. The Varian Eclipse Version 7.3.10^®^ treatment planning system was utilized.

### Statistical analysis

Follow-up was estimated using the reverse Kaplan-Meier method. Recurrence-free survival (RFS) and overall survival (OS) were estimated using the Kaplan-Meier method. Survival rates were defined as the time spam between the beginning of treatment and the first event. Survival curves were compared using the log-rank test for the univariate analysis.

Statistical analyses were performed using the SPSS software, version 18.0 (SPSS Inc.; Chicago, USA). P-values lower than 0.05 were considered to be statistically significant.

## RESULTS

### Overall survival

Median follow-up was 36.6 months (3-110 months). Median age was 54.4 years (34-81 years) and 142 patients were male (98.6%). The reviewed patient and tumor characteristics are shown on Table 1.

**Table 1:** Patient and tumor characteristics

	Total	Surgery	Radical
	population	group	RT group
	(*n* = 144)	(*n* = 63)	(*n* = 81)
Median age (years)	54.4	55.0	52.0
Gender			
Male	142 (98.6%)	62 (98.4%)	80 (98.8%)
Female	2 (1.4%)	1 (1.6%)	1 (1.2%)
Location			
Piriform synus	117 (81.3%)	57 (90.5%)	60 (74.1%)
Posterior wall	8 (5.6%)	3 (4.8%)	5 (6.2%)
Post cricoide	2 (1.4%)	0	2 (2.5%)
Not otherwise specified	17 (11.8%)	3 (4.8%)	14 (17.3%)
Disease stage			
III	35 (24.3%)	13 (20.6%)	22 (27.2%)
IV	109 (75.7%)	50 (79.4%)	59 (72.8%)
Induction CT	26 (18.1%)	1 (1.6%)	25 (30.9%)
Concomitant CT	112 (77.8%)	39 (61.9%)	73 (90.1%)
Enteral feeding tube	47 (32.6%)	11 (17.5%)	36 (44.4%)
Tracheostoma	69 (47.9%)	32 (50.8%)	37 (45.7%)

CT, chemotherapy; RT, radiotherapy

Median OS for the entire population was 26 months (49 months for stage III and 21 months for stage IV).

Two-year and five-year OS was 51.0% (59.9% for stage III and 48.2% for stage IV), and 30.5% (39.2% for stage III and 27.7% for stage IV), respectively.

**Figure 1:**
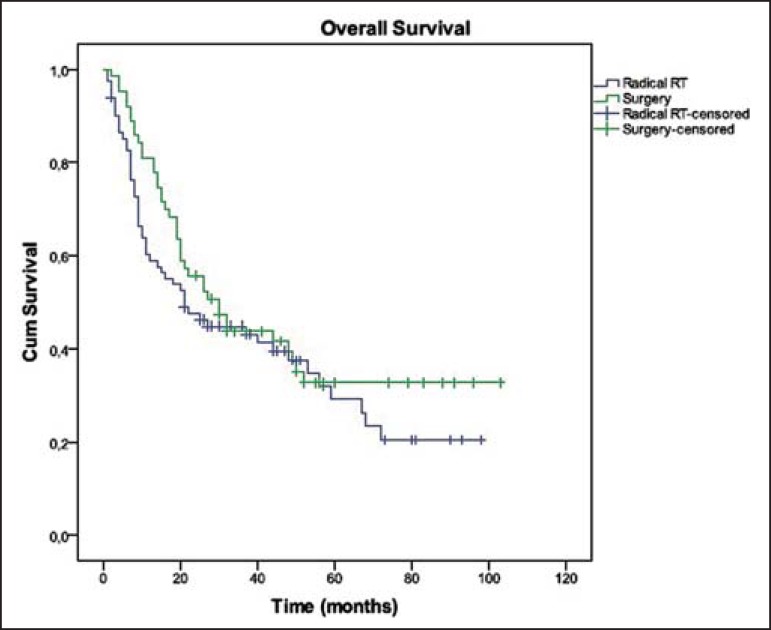
Overall survival in both groups.

The treatment regimen had no significant impact in OS (Figure 1; Table 2).

**Table 2:** Overall survival in both groups

	Surgery group	Radical RT group	
	(*n* = 63)	(*n* = 81)	
Median overall survival	30 months	21 months	p = 0.274
2-year overall survival	55.6%	47.5%	
5-year overall survival	32.8%	29.2%	

RT, radiotherapy

### Recurrence-free survival

Overall, 51 patients (35.4%) had relapsed either locally, regionally or at the metastatic level, and 17 patients (11.8%) never achieved complete response (persistent disease).

The majority of the cases of relapse were loco-regional (n = 28; 54.9%); 18 local recurrences; 6 neck recurrences and 4, both local and regional recurrence. Distant metastases were identified in 24 patients (47.1%), 6 of whom had both loco-regional recurrence, and one, distant relapse.

Median RFS for the entire population was 18 months (22 months for stage III, and 18 months for stage IV).

Two-year and five-year RFS was 42.8% (46.7% for stage III and 41.5% for stage IV), and approximately 28.5% (35.0% for stage III and 26.6% for stage IV), respectively.

**Figure 2:**
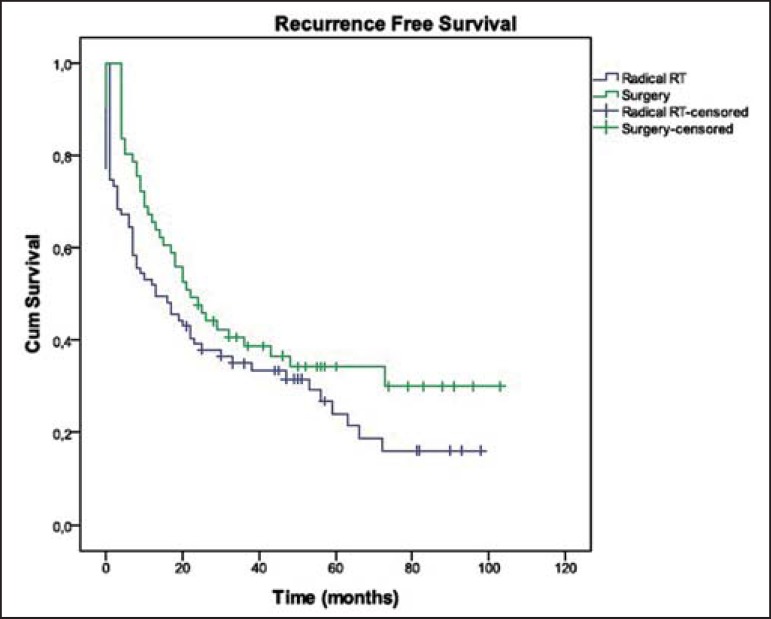
Recurrence free survival in both groups.

The treatment regimen had no significant impact on the RFS (Figure 2; Table 3).

**Table 3:** Recurrence free survival in both groups

	Surgery	Radical RT	
	group	group	
	(*n* = 63)	(*n* = 81)	
Median recurrence free survival	22 months	13 months	p = 0.126
2-year recurrence free survival	47.5%	39.1%	
5-year recurrence free survival	~31.0%	24.1%	

RT, radiotherapy

## DISCUSSION

As previously mentioned, hypopharyngeal cancer has the worst prognosis of any head and neck cancer. Considering that this entity constitutes only 3-5% of all head and neck tumors, treatment outcomes reported in the literature are frequently referred to hypopharynx in combination with larynx cancer or other cancers of head and neck sub-sites^(2)^. Probably, this is not the most appropriate way to report them, since it might overestimate outcomes associated with tumors in this particular location. Den et al.^(3)^ have investigated whether patients with hypopharynx cancer performed as well as oropharyngeal primaries in the definitive setting, or as larynx primaries in the post-operative setting, in the Radiation Therapy Oncology Group (RTOG) trials^(4,5)^. According to such analysis, stratification by disease site should be considered in future multi-head and neck trials.

The expected 2-year OS in hypopharyngeal SCC is around 50% in disease stage III and 30% in stage IV. Five-year OS ranges from 35% in stage III to 18% in stage IV^(6,7)^. In the present series, 2-year OS was 59.9% for stage III and 48.2% for stage IV; and 5-year OS was 39.2% for stage III and 27.7% for stage IV. The apparently better results in the present study may be due to the small sample size and to other hidden selection biases that could not be excluded due to the retrospective nature of the study. Nevertheless, the authors consider these values consistent with the expectable outcomes.

As regards the therapeutic strategy (surgery vs. radical RT), there were no significant differences between both groups with respect to survival outcomes. Only a tendency towards better OS (55.6% vs. 47.5% at 2 years; 32.8% vs. 29.2% at 5 years) and RFS (50.0% vs. 41.7% at 2 years; 13.3% vs. 12.5% at 5 years) was perceptible in the group submitted to surgery followed by adjuvant RT, but without a significant impact. At this point, the authors emphasize the complexity of a direct comparison of surgically treated patients with radiation treated patients, particularly in a retrospective designed study like the present one. Such a difficulty is associated with common selection bias whereby some patients are selected for surgery and others for RT. In the advanced stage setting, concurrent RT/CT is frequently proposed for patients with low to moderate volume disease in which functional status has not been irreversibly compromised. Otherwise, a primary surgical approach followed by postoperative RT is typically adopted. In the present study, there was, in fact, a significant heterogeneity with respect to the T stage and N stage (stage III: 5 patients with stage T1-2N1 and 30 patients with T3N0-1; stage IV: 30 patients with stage T1-3N2, 18 patients with stage T1-3N3, 14 patients with stage T4N0; and 47 patients with stage T4N1-3) complicating even more this comparison. Even so, the results observed in the present series are in line with the ones reported by other authors^(8,9)^.

In the study developed by Huang et al.^(8)^, RT/CT using IMRT has revealed comparable results with primary surgery in terms of therapeutic outcomes, and provided a 40% larynx preservation five-year survival rate.

In a matched-pair analysis by Rades et al.^(9)^, including T3/4 larynx and hypopharynx cancer, RT/CT did not result in significantly worse outcomes in terms of loco-regional management, metastasis-free survival and OS, but in a considerably higher rate of larynx preservation as compared with the primary surgical approach.

In relation to the sequential approach, the randomized phase III trial by EORTC Head and Neck Group compared induction CT followed by RT with surgery followed by RT^(1)^. Such study included 194 patients with pyriform sinus cancer, and compared surgery plus 50-70 Gy of adjuvant RT with patients who responded to induction CT with cisplatin + 5-fluorouracil and received 70 Gy of RT following three courses of CT. Median survival times were 25 months in the surgery plus RT group and 44 months in the CT plus RT group (*p* = 0.006). Loco-regional management rates were similar in both groups, whereas distant failure was more common in the surgery plus RT group (36% vs. 25%; *p* = 0.041).

Other retrospective studies have reported comparable survival for advanced hypopharynx cancer treated with induction CT followed by definitive RT with that achieved with the surgical approach and postoperative RT^(10,11)^.

Kim et al.^(12)^ have compared the treatment results of locally advanced hypopharyngeal carcinoma according to treatment modalities, and considered that nonsurgical therapy (induction CT plus RT) is an effective strategy to achieve organ preservation without compromising the survival of locally advanced hypopharyngeal carcinoma patients.

Considering that larynx preservation is such an important issue, it would be desirable to develop a randomized trial focused on the comparison of surgery followed by RT vs. concurrent (instead of sequential) RT/CT. No such a trial has been developed so far, and some difficulties could be expected in the creation of such a trial. The rarity of this disease, and the time required for data collection explains in part the absence of a randomized trial undertaken to evaluate the role played by RT/CT in the treatment of advanced hypopharyngeal cancer. Additionally, patients longing for preserving their larynx probably will not be willing to participate in a randomized clinical trial offering a 50% chance of larynx preservation, as a non-surgical option is available.

An accurate understanding of prognostic factors is important to select the optimal treatment for the individual patient or to stratify patients for clinical trials or statistical analyses. Total tumor volume is a well known prognostic parameter that impacts on the loco-regional management and OS. Such a factor should be taken into account for the selection of high-risk patients and for their stratification in clinical trials or statistical analyses, as suggested by Rudat et al.^(13)^.

Despite the absence of prospective data, definitive RT/ CT does not seem to be inferior to surgery plus RT for locally advanced hypopharynx cancer, and allows for a significant higher larynx preservation rate. Additionally, it is possible that the results of definitive RT/CT are further enhanced by the use of hyperfractionated RT instead of conventional fractionation^(14-16)^, and by the utilization of positron emission tomography/computed tomography in the RT planning (a possible way to implement biological adaptive radiotherapy)^(17)^. Another way to optimize these results would be the systematic use of more effective CT regimens such as docetaxel + cisplatin + 5-fluorouracil.

## CONCLUSIONS

In summary, the survival outcomes for locally advanced hypopharynx cancer in the present study are consistent with other studies in the literature.

The treatment approach had no significant impact on OS and RFS, as described in other series. Given the equivalence between these two strategies suggested in several series, definitive RT/CT seems to be an advantageous option as it improves organ preservation and, consequently, the functional outcome.

Recent developments in radiation treatment techniques, together with the introduction of effective concurrent CT regimens, could hopefully increase tumor management and survival rates.

Because of the retrospective nature of the present study and the relatively small number of patients, the presently described findings cannot be considered conclusive.
